# Sleep Quality, Daytime Sleepiness and Sleep Hygiene Among Youth in a Rural District in South India: A Cross-Sectional Study

**DOI:** 10.7759/cureus.69551

**Published:** 2024-09-16

**Authors:** Neethu George, Lloyds Earnesteen, Meera George, Rock B Dharmaraj, Neeraj V Mohandas, Vijay Anand V, Tamilarasan Muniyapillai, Adarsh E Chacko, Karthikeyan Kulothungan

**Affiliations:** 1 Community Medicine, Dhanalakshmi Srinivasan Medical College and Hospital, Siruvachur, Perambalur, IND; 2 Psychiatry, Government Sivagangai Medical College, Sivagangai, IND; 3 Community Medicine, Family Health Centre, Kumily, Kumily, IND

**Keywords:** daytime sleepiness, pittsburgh sleep quality index, quality of sleep, the epworth sleepiness scale, youth health

## Abstract

Background

Adequate sleep is crucial for youth cognitive function, academic performance, and mental health. However, various factors, including academic pressure, technology use, and socio-cultural norms can significantly impact sleep patterns, particularly in rural settings. This cross-sectional study assessed sleep quality, daytime sleepiness prevalence, and sleep hygiene practices among youth in a rural South Indian district. We also investigated factors associated with these sleep parameters in this understudied population

Methods

This was a cross-sectional study among 852 young subjects who were assessed with a self-reported proforma of socio-demographic details, behavioural factors, Pittsburgh Sleep Quality Index and Epworth Sleepiness Scale. Regression model was created to analyse the predictor for sleep quality and daytime sleepiness with respect to sociodemographic variables, behavioural factors and sleep hygiene practices.

Results

Our study revealed that 49.4% (n=421) of participants exhibited poor sleep quality, while 29.5% (n=251) reported abnormal daytime sleepiness. The most prevalent sleep hygiene practices were reading in bed (68.5%, n=584) and pre-bedtime eating (56.22%, n=479). Multivariate analysis indicated that sleep quality was significantly associated with accommodation type, with increased odds for those in private accommodation (adjusted odds ratio (AOR) 1.29, 95% CI: 1.14-1.62) and hostels (AOR 3.79, 95% CI: 1.79-8.06). Additionally, eating in bed (AOR 1.42, 95% CI: 1.03-1.95) and pre-bedtime eating (AOR 1.41, 95% CI: 1.07-1.88) were also associated with poor sleep quality. Factors significantly associated with daytime sleepiness included younger age (AOR 0.84, 95% CI: 0.75-0.94), non-medical academic streams (AOR 1.94, 95% CI: 1.33-2.83), extensive internet usage (three or more hours) (AOR 1.87, 95% CI: 1.15-3.13), watching TV in bed (AOR 1.46, 95% CI: 1.06-1.99), writing (AOR 1.45, 95% CI: 1.02-2.06), and eating in bed (AOR 1.55, 95% CI: 1.09-2.21).

Conclusion

This study reveals a significant incidence of poor sleep quality and daytime drowsiness among young individuals residing in rural areas of South India. The results of our study emphasize significant connections between sleep disturbances and several modifiable aspects, such as the kind of accommodation, eating habits, and use of technology. The impact of eating behaviours, both in bed and before bedtime, on sleep quality and daytime sleepiness underscores the importance of proper sleep hygiene education. Furthermore, the relationship between extensive internet usage and daytime sleepiness points to the growing influence of digital technology on youth sleep patterns. These findings emphasize the need for comprehensive sleep health programs tailored to rural youth. Such initiatives should address environmental factors, promote healthy sleep hygiene practices, and provide guidance on balanced technology use. Additionally, the varying impact of academic streams on sleep parameters suggests that sleep health strategies may need to be customized for different educational contexts.

## Introduction

*"Sleep-the healthy spa that rejuvenates the body and its functions, pampering therapy to relax, renew, and refresh”. *- Rock Britto.

Youth, defined as individuals aged 15 to 24, constitute approximately 30% of India's population [1]. This demographic is undergoing significant physiological and psychosocial transitions, including changes in sleep patterns and brain development [2]. Adolescents and young adults typically experience a biological delay in sleep onset, known as sleep phase delay, due to alterations in the circadian timing system and homeostatic sleep-wake processes [3].

The adolescent brain undergoes substantial maturational changes, partially influenced by pubertal hormones. Sex steroids affect neurogenesis, synaptic organization, receptor expression, and neurite outgrowth, consequently impacting internal sleep processes [4]. Moreover, academic and social demands often exacerbate sleep challenges in this population.

Contemporary lifestyle factors and environmental conditions are increasingly influencing youth sleep patterns, potentially compromising sleep hygiene, quality, and leading to excessive daytime sleepiness. Sleep quality, encompassing various aspects of sleep experience [5], is crucial for cognitive function, mood regulation, and overall health. Poor sleep quality can result in daytime fatigue, cognitive impairment, and mood alterations, potentially leading to long-term health consequences [5,6].

Sleep hygiene refers to behavioral and environmental practices that promote healthy sleep [7,8]. Inadequate sleep hygiene can lead to reduced sleep quantity and quality, resulting in both short-term (e.g., daytime sleepiness, attention deficits) and long-term consequences (e.g., cardiovascular problems, metabolic disorders) [9].

While previous studies have independently examined sleep quality, quantity, and hygiene among youth [10-12], there is a need for comprehensive research that integrates these aspects, particularly in the Indian context. This study aims to assess sleep quality, prevalence of daytime sleepiness, and sleep hygiene practices among youth in a rural district of South India. Additionally, the study seeks to identify factors influencing sleep quality and daytime sleepiness in this population.

## Materials and methods

Study setting and study participants

The study was conducted as a cross-sectional investigation among late adolescents and young adults (aged 18-25 years) affiliated with a private educational institution in a rural district of Tamil Nadu, a south Indian state, over a two-month period from July to September 2023. The study encompassed students from diverse academic streams, including engineering, medicine, dental, and management programs, to provide a comprehensive perspective on sleep patterns across various disciplines.

Data collection was facilitated through a digital platform, with the study questionnaire uploaded to Google Forms and initially distributed to contacts of the investigators. To maximize participation and ensure representation across all targeted academic streams, a snowball sampling technique, a form of non-probability sampling, was employed. This method was strategically chosen to leverage existing networks within each academic stream, allowing for efficient dissemination of the study tool. Subjects who self-reported being diagnosed with any form of sleep disorder or having medications for chronic medical conditions were excluded from the study through specific screening questions incorporated into the Google Forms questionnaire. The snowball sampling approach began with the identification of initial participants in each stream (engineering, medicine, dental, and management). These participants were then encouraged to share the questionnaire link with their peers in the same academic program. This networking effect allowed the study to rapidly expand its reach within each discipline, gathering a substantial number of responses from each stream.

Ethics committee approval 

Ethics committee approval was obtained from the institution's ethics committee (DSMCH-352B, 18/07/2023) and informed consent was taken before the start of the study. Non-formal permission from the concerned subject departments was also taken before the study. This clinical research was done following the ethical principles for medical research involving human subjects in accordance with the Helsinki Declaration of 2013.

Sample size

The investigator disseminated the study tools through web-based platforms for a period of two months. The investigators adopted a saturation sampling strategy, aiming to include as many eligible participants as possible from the accessible student population. Post hoc power analysis was performed upon completion of data collection to assess the statistical power of the study based on the achieved sample size. The power of the study with 852 samples on proportion of sleep quality showed 93.3%, with a precision of 5% and 95% confidence interval.

Study tools

The questionnaire (Appendix 1) consisted of sociodemographic details, Epworth Sleepiness Scale and Pittsburgh Sleep Quality Index (PSQI) and a sleep hygiene questionnaire.

Socio-demographic questions including age, gender, number of family members, marital status, place of stay, and education of father and mother were included. Behavioural factors including the time spent online per day, subjective feeling of depressed mood, and academic performance were noted.

Epworth Sleepiness Scale is a standardized tool used to measure daytime sleepiness. It contains eight questions, each scoring 0-3 with increasing numbers signifying a higher chance of “dozing” while engaged in specific activities of daily life. A score of less than 10 is generally considered clinically normal [13].

Pittsburgh Sleep Quality Index consists of 19 items and measures several different aspects of sleep, offering seven component scores and one composite score. The component scores consist of subjective sleep quality, sleep latency (i.e., how long it takes to fall asleep), sleep duration, habitual sleep efficiency (i.e., the percentage of time in bed that one is asleep), sleep disturbances, use of sleeping medication, and daytime dysfunction. Each item is weighted on a 0-3 interval scale. The global PSQI score is then calculated by totaling the seven component scores, providing an overall score ranging from 0 to 21, where lower scores denote a healthier sleep quality. A global score of 5 or more indicates poor sleep quality, the higher the score, the worse the quality [14].

The sleep hygiene questionnaire included a few questions regarding subject habits revolving around bed or immediately before/during bedtime like eating, reading, writing and entertainment.

To ensure data integrity, email addresses were collected to prevent duplicate submissions. A total of 860 forms were received, of which eight were excluded from the final analysis due to incomplete demographic information or sleep hygiene questionnaire responses, resulting in 852 valid submissions for the study

Statistical analysis

The filled responses were collected, and Excel sheets (Microsoft, Redmond, WA, USA) were imported from the Google sheets and analysed using SPSS Version 23.0 (IBM Corp., Armonk, NY, USA). Descriptive statistical analysis was performed on sociodemographic variables including age, gender, year of study, and parents' education/occupation. For continuous variables such as age, measures of central tendency (mean and median) and dispersion (standard deviation and interquartile range) were calculated if required. For categorical variables like gender and year of study, frequencies and percentages were computed. Sleep quality and daytime sleepiness were categorized based on standardized cut-off scores, with the distribution of participants across these categories presented in tables and figures. The sleep hygiene practices were also represented as categories. The bivariate analysis was done using independent t-test and chi-square test. A multivariable logistic regression analysis was conducted to identify predictors of sleep quality and daytime sleepiness. Variable selection was performed using a purposeful selection method. Initially, all variables with a significance level of p < 0.20 in bivariate analyses were considered for inclusion in the multivariable model. The model included sociodemographic variables (age, gender, stream of study, marital status, place of stay), behavioral factors (social media usage, reported academic performance), and sleep hygiene practices. The model's goodness-of-fit was evaluated using the Hosmer-Lemeshow test, with p > 0.05 indicating adequate fit. Odds ratios with 95% confidence intervals were calculated for each predictor in the final model.

## Results

Sociodemographic details

The study was done among 852 subjects between the age of 18-25 years. The mean (SD) age in years was 21.61 (1.45) years. In the study 548 (64.3%) were females and 304 (35.7%) were males. In the study, 98 (11.5%) are studying engineering courses, 39 (4.6%) are studying dental courses, 93 (10.9%) are studying for a degree in arts and science colleges, 13 (1.5%) are studying law-related courses, 13 (1.5%) are studying management studies, 559 (65.6%) are studying for a medical degree and 37 (4.3%) are studying other courses like nursing and pharmacy courses. So, 221 (25.9%) subjects were from non-medical related courses.

In the study, 371 (43.5%) stay at home, 441 (51.8%) stay at hostel and 40 (4.7%) stay at private accommodation. In parent’s education, 368 (43.2%) of their fathers are graduates, and 375 (44%) of their mothers are graduates. Among the subjects, 38 (4.5%) spent one hour per day, 85 (10%) spent two hours per day, 167 (19.65%) spent three hours per day, 192 (22.5%) spent four hours per day, 370 (43.4%) spent five hours per day in social media. In the study 777 (91.2%) are not on any medication while 75 (8.8%) are on some medication for diseases like thyroid disorders, respiratory disorders, migraine etc. In the study 706 (82.9%) had reported an average performance in academics, and 89 (10.4%) reported excellent and rest as poor.

Sleep quality

Table 1 shows the description of the Pittsburgh Sleep Quality Index and its various components. The study showed that by assessing the subjects based on the Pittsburgh Sleep Quality Index, 431 (50.6%) students had good sleep quality (score<5) and 421 (49.4%) students had poor sleep quality scores (>5).

**Table 1:** Descriptive Statistics of Pittsburgh Sleep Quality Index (PSQI) Scores (n=852)

Categories	Mean±SD	Min–Max
Global PSQI score (total score)	5.92±3.13	0–21
Comp. 1: subjective sleep quality	0.96±0.84	0–3
Comp. 2: sleep latency	1.14±.993	0–3
Comp. 3: sleep duration	0.95±0.92	0–3
Comp. 4: habitual sleep efficiency	0.62±.95	0–3
Comp. 5: sleep disturbances	1.06±0.53	0–3
Comp. 6: use of sleeping medications	0.32±0.74	0–2
Comp. 7: daytime dysfunction	0.88±0.81	0–3

Daytime sleepiness

The daytime sleepiness was assessed by the Epworth Sleepiness Scale where 601 (70.5%) students had no sleep disorder (score less than 10) and 251 (29.5%) students had presence of abnormal daytime sleepiness (score more than or equal to 10).

Sleep hygiene practices

**Figure 1:**
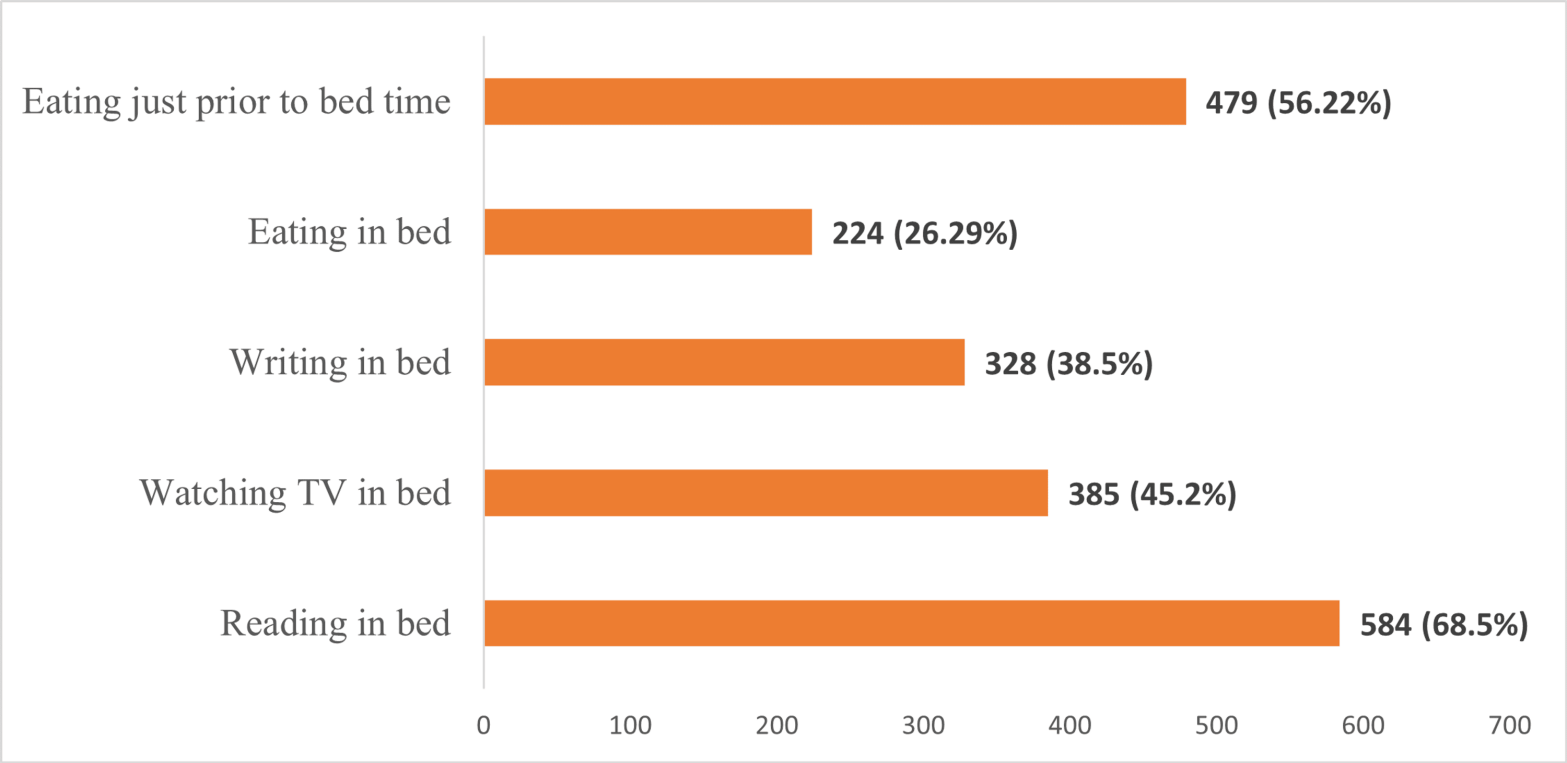
Frequency (%) of subjects with the following sleep hygiene practices (n=852) The figure shows the proportion of students who responded "Yes" to the following questions

Figure 1 shows the frequency (%) of various sleep hygiene practices the subjects followed.

Out of a total of 852 students, 65 (7.6%) reported going to bed before 10 PM, 220 (25.8%) between 10 and 11 PM, 234 (27.5%) between 11 PM and 12 AM, and 333 (39.1%) after 12 AM during weekends. During weekdays, 333 students (39.1%) went to bed between 11 PM and 12 AM, while 50 students (5.9%) went to bed before 10 PM. Among the 852 students, 350 (41.1%) reported waking up between 6 and 7 AM on weekdays, 256 (30%) between 7 and 8 AM, and 111 (13%) after 8 AM. On weekends, 372 students (43.7%) reported waking up after 8 AM, 253 (29.7%) between 7 and 8 AM, and 47 (5.5%) before 6 AM. Regarding snoring habits, 718 students (84.3%) reported never snoring, 50 (5.9%) reported snoring daily, 48 (5.6%) reported snoring once a week, and 36 (4.2%) reported snoring once a month.

Regression model predicting sleep quality

Table 2 shows the regression model to assess predictor variables for sleep quality. The bivariate analysis was done using independent t-test and chi-square test. The variables with p-value <0.2 were taken into regression analysis. The goodness-of-fit of the logistic regression model was assessed using the Hosmer-Lemeshow test. The test yielded a chi-square statistic of 6.29 with a corresponding p-value of 0.62. This result suggested the model is fit. The multivariable logistic regression model was statistically significant, χ2(4) = 29.72 p=0.001. The model explained 4.6% (Nagelkerke R2) of the variance in PSQI and correctly classified 50.6% of cases. The table showed that people who stayed in private accommodation and hostel had 3.79 times (p<0.001) and 1.29 (p=0.02) times chance of having poor sleep quality in comparison to those staying at home. Also, students who eat in bed and eat prior to bedtime have a 1.42 (p=0.025) and 1.41 (p=0.03) times chance of having poor sleep quality.

**Table 2:** Multivariable logistic regression predicting poor sleep quality * p-value<0.05, # p-value<0.001 a: Independent t-test (mean (standard deviation)), All others: Chi-square test (n (%)) Multivariable logistic regression of all variables with good sleep quality as reference category Reference category: Sex- female, Stream- medical, Marital status- married, Place of stay- home, internet usage- ≤2 hours, reported academic excellence- poor, Reading in bed- No, Watching TV in bed- No, Writing in bed- No, eating in bed- No, eating prior to bedtime- No

Basic Characteristics		Pittsburgh sleep quality		Adjusted odds ratio	95% Confidence interval
		Good	Poor		
Age of the student in years ^a^		21.61 (1.41)	21.62 (1.48)	0.99	0.91,1.10
Sex	Female	269 (49.1%)	279 (50.9%)	-	
	Male	162 (53.3%)	142 (46.7%)	0.78	0.58, 1.04
Stream	Medical	326 (51.7%)	305 (48.3%)	-	
	Non-medical	105 (47.5%)	116 (52.5%)	1.3	0.92, 1.84
Marital status	Married	12 (60%)	8 (40%)	-	
	Unmarried	419 (50.4%)	413 (49.6%)	1.7	0.67, 4.35
Place of stay	Home	198 (53.4%)	173 (46.6%)	-	
	Hostel	223 (50.6%)	218 (49.4%)	1.29	1.14, 1.62*
	Private accommodation	10 (25%)	30 (75%)	3.79	1.79, 8.06#
Internet usage per day in hours	≤2	66 (53.7%)	57 (46.3%)	-	
	≥3	365 (50.1%)	364 (49.9%)	1.71	0.79, 1.73
Reported academic performance	Poor	27 (47.4%)	30 (52.6%)	-	
	Average	359 (50.8%)	347 (49.2%)	0.83	0.48, 1.46
	Excellent	45 (50.6%)	44 (49.4%)	0.84	0.42, 1.66
Reading in bed	No	148 (55.2%)	120 (44.8%)	-	
	Yes	283 (48.5%)	301 (51.5%)	0.84	0.61, 1.16
Watching TV in bed	No	235 (50.3%)	232 (49.7%)	-	
	Yes	196 (50.9%)	189 (49.1%)	1.16	0.87, 1.55
Writing in bed	No	276 (52.7%)	248 (47.3%)	-	
	Yes	155 (47.3%)	173 (52.7%)	1.01	0.74, 1.39
Eating in bed	No	336 (53.5%)	292 (46.5%)	-	
	Yes	95 (42.4%)	129 (57.6%)	1.42	1.03, 1.95*
Eating prior to bedtime	No	208 (55.8%)	165 (44.2%)	-	
	Yes	223 (46.6%)	256 (53.4%)	1.41	1.07,1.88*

Regression model predicting daytime sleepiness

Table 3 shows the regression model to assess predictor variables for daytime sleepiness. The bivariate analysis was done using independent t-test and chi-square test. The variables with p-value <0.2 were taken into regression analysis. The goodness-of-fit of the logistic regression model was assessed using the Hosmer-Lemeshow test. The test yielded a chi-square statistic of 11.08 with a corresponding p-value of 0.20. This result suggested the model is fit. The multivariable logistic regression model was statistically significant, χ2(4)=56.72, p<0.001. The model explained 9.2% (Nagelkerke R2) of the variance in daytime sleepiness and correctly classified 70.5% of cases. The table showed that as age increases the odds of experiencing abnormal daytime sleepiness decrease by 16% (p=0.018). Subjects who belong to non-medical streams have a 1.94 times (p<0.001) chance of having abnormal daytime sleepiness in comparison with that of the medical stream. Subjects who use the internet for three or more hours had a 1.87 times (p<0.001) chance of having abnormal daytime sleepiness in comparison with that of internet users two or fewer hours per day. Subjects who watch TV, write and eat in bed had 1.46 (p=0.03), 1.45 (p=0.26) and 1.55 (p<0.001) times chance of having abnormal daytime sleepiness in comparison to those people who do not perform these activities.

**Table 3:** Multivariable logistic regression model predicting abnormal daytime sleepiness * p-value<0.05, # p-value<0.001 a: Independent t-test (mean (standard deviation)), All others: Chi-square test (n (%)) Multivariable logistic regression of all variables with normal daytime sleepiness as reference category Reference category: Sex- female, Stream- medical, Marital status- married, Place of stay- home, internet usage- ≤2 hours, reported academic excellence- poor, Reading in bed- No, Watching TV in bed- No, Writing in bed- No, eating in bed- No, eating prior to bedtime- No

Basic Characteristics		Daytime sleepiness		Adjusted odds ratio	(95% CI)
		Normal	Abnormal		
Age of the student in years ^a^		21.72 (1.48)	21.36 (1.34)	0.84	0.75, 0.94*
Sex	Female	379 (69.2%)	169 (30.8%)		
	Male	222 (73%)	82 (27%)	0.82	0.59, 1.15
Stream	Medical	461 (73.1%)	170 (26.9%)	-	
	Non-medical	140 (63.3%)	81 (36.7%)	1.94	1.33, 2.83#
Marital status	Married	18 (90%)	2 (10%)	-	
	Unmarried	583 (70.1%)	249 (29.9%)	3.36	0.76, 14.87
Place of stay	Home	259 (69.8%)	112 (30.2%)	-	
	Hostel	311 (70.5%)	130 (29.5%)	1.21	0.85, 1.71
	Private accommodation	31 (77.5%)	9 (22.5%)	0.84	0.38, 1.86
Internet usage per day in hours	≤2	98 (79.7%)	25 (20.3%)	-	
	≥3	503 (69%)	226 (31%)	1.87	1.15, 3.13#
Reported academic performance	Poor	36 (63.2%)	21 (36.8%)	-	
	Average	504 (71.4%)	202 (28.6%)	1.48	0.71, 3.07
	Excellent	61 (68.5%)	28 (31.5%)	0.88	0.54, 1.45
Reading in bed	No	197 (73.5%)	71 (26.5%)	-	
	Yes	404 (69.2%)	180 (30.8%)	1.06	0.73, 1.53
Watching TV in bed	No	351 (75.2%)	116 (24.8%)	-	
	Yes	250 (64.9%)	135 (35.1%)	1.46	1.06,1.99*
Writing in bed	No	391 (74.6%)	133 (25.4%)	-	
	Yes	210 (64%)	118 (36%)	1.45	1.02, 2.06*
Eating in bed	No	465 (74%)	163 (26%)	-	
	Yes	136 (60.7%)	88 (39.3%)	1.55	1.09, 2.21#
Eat prior to bedtime	No	269 (72.1%)	104 (27.9%)	-	
	Yes	332 (69.3%)	147 (30.7%)	1.01	0.74, 1.39

## Discussion

The study was done with an aim to measure the assessment of sleep (quality, prevalence of daytime sleepiness and hygiene practices) and to measure the factors that influence the sleep quality and prevalence of daytime sleepiness.

Prevalence of poor sleep quality

The study showed that by assessing the subjects based on PSQI, 421 (49.4%) students had poor sleep quality. In studies done in Orissa [15], Kerala [12] and Karnataka [16], 45%, 37.6% and 55.3% had poor sleep quality, respectively. In a study done in Nepal, 38.2% of undergraduate medical students and 35.4% of non-medical students had poor sleep quality [17,18]. In a study done in Mauritius, 30.7% of youth had poor sleep quality with alcohol consumption and tobacco smoking after 18:00 hours, pre-bedtime awakening actions, and a poor sleep environment [19]. In a study done in 2020, 44.23% had poor sleep quality among medical students, where 39.8% and 48.2% of male and female students had poor sleep quality [20]. In this study, 33.7% and 66.3% of male and female students had poor sleep quality. In a study done in Brazil, 39.5% had worse sleep quality [21]. The variation in the proportion of sleep quality across different studies can be attributed to a range of factors. Differences in population demographics, such as age, gender, and health status, can significantly influence sleep quality, leading to diverse findings. Additionally, the use of varying assessment methods and tools, as well as different definitions and criteria for sleep quality, can result in inconsistent results between studies. Cultural factors and lifestyle habits, which vary widely across populations, also play a crucial role in shaping sleep patterns. Environmental conditions, such as noise levels, light pollution, and living conditions, can further impact sleep quality. Moreover, the sample size and selection criteria used in different studies can lead to discrepancies, with smaller or less representative samples potentially causing greater variation. Socioeconomic factors, including stress levels, work schedules, and access to healthcare, also contribute to the differences in sleep quality observed across studies.

Determinants of sleep quality

This study showed that the stay of people in private accommodations and hostels turned out to be an influencing factor in sleep quality. Similar results were seen in studies where students living by themselves were associated with poor sleep quality [22,23]. The reason for this may be due to young people accumulating stress from tending needs on their own, along with sleep disturbances, which would have resulted in poor sleep quality. In this study, age differences were not significant, which can be due to the narrow age gap between the subjects. Also, the study showed sleep hygiene practices like eating in bed and eating prior to bedtime negatively affected sleep quality. Various studies [8,21] have shown similar results where meal timing and other hygiene practices reduce the quality of sleep, particularly sleep latency. A study done in Iran showed that eating heavy meals, smoking adjacent to bedtime, and performing dynamic physical activity before bedtime made the subjects acquire poor sleep quality [8]. The studies have shown that sleep hygiene practices overall have a positive influence on sleep quality, which shows the need for a separate environment for sleep apart from routine activities. The environment should be well utilized for only sleep with emphasis to lifestyle interventions at the right time.

Daytime sleepiness prevalence by Epworth Sleepiness Scale

The daytime sleepiness was assessed by Epworth Sleepiness Scale. In the study, 251 (29.5%) students have presence of abnormal daytime sleepiness. A study done in India showed the prevalence of excessive daytime sleepiness as 44.5% [24]. Studies done in Maharashtra and Karnataka showed a prevalence of 17.3% and 47.4% of excessive daytime sleepiness respectively [25,26]. In a study in Mauritius, 32.7% had abnormal daytime sleepiness [19]. According to a study done in Saudi Arabia, 21.1% of subjects had abnormal daytime sleepiness [27]. In a study done in Ethiopia, 31.07% was the prevalence with association with depression and other sleep disorders [28]. The variation in the prevalence of daytime sleepiness among various studies can be attributed to disparities in study populations, sample sizes, assessment methodologies, cultural influences, lifestyle variations, and divergent definitions or criteria employed to quantify daytime sleepiness. Furthermore, changes in these aspects could potentially be influenced by environmental factors, including climate and socioeconomic conditions.

Factors affecting daytime sleepiness

In the study people who are of lower age, subjects who belong to the medical stream and those using the internet for three or more hours had a chance of having abnormal daytime sleepiness. Usually, age factor makes a positive relation between daytime sleepiness due to the increased demands of work or health habits. In this study people with slightly lower age had excessive daytime sleepiness which showed the possibility of affection of sleep with other factors predominant among youth people like metabolic factors and mood factors [29]. The overuse of screens causes sleep-related issues including staying up later, taking longer to fall asleep, waking up more frequently during the night, having trouble waking up, and feeling sleepy during the day [30]. Also increased prevalence in the medical stream can be due to varied study hours with demanding curriculum. Sleep hygiene practices like watching TV in bed, writing in bed and eating in bed had a negative influence on daytime sleepiness. Studies done in various parts of the world showed that sleep hygiene practices have a significant association with abnormal daytime sleepiness [24,27]. 

Strengths

This cross-sectional study offers valuable insights into the sleep habits and hygiene practices of rural South Indian youth, filling a notable void in the existing literature. The main advantages of this study are its emphasis on a population that has received less attention and the utilization of validated measures to evaluate the quality of sleep and daytime drowsiness. The study's comprehensive methodology, which investigates various aspects of sleep health, improves our comprehension of sleep-related problems in this specific population.

Limitations

The use of a cross-sectional design prevents the establishment of causal links between variables. The utilization of non-probability sampling constrains the extent to which findings can be applied to settings other than the specific one under study. The use of web-based platforms for data collecting may have led to response bias, including social desirability bias, sample bias due to inaccurate submissions, and possibly fatigue-induced replies. Self-reported data is susceptible to recall bias. The study's limitation to a particular rural district narrows its applicability to other regions or metropolitan environments. Furthermore, the absence of objective sleep assessments, such as actigraphy or polysomnography, implies that sleep patterns were not independently confirmed.

Recommendations

Academic institutions ought to provide extensive sleep instruction programs specifically designed to meet the needs of young adults. These programs should not only cover the basics of sleep, but also explore its relationships with stress management, technology usage, nutrition, and physical activity. The authors suggest creating a standardized module called "Effective Sleep Hygiene Practices" that is like commonly used health protocols. This module would offer straightforward, step-by-step guidance to establishing healthy sleep habits. This project should prioritize the significance of sleep etiquette acquired during childhood, providing help on preserving and adjusting these habits throughout adolescence and early adulthood. Also steps are made to cultivate a culture of optimal sleep patterns among young adults, with the goal of enhancing academic performance, improving stress coping abilities, and fostering general wellness.

## Conclusions

The study demonstrates substantial sleep health issues among young individuals in rural South India, with more than 50% having poor sleep quality and close to a third reporting abnormal daytime sleepiness. Accommodation type is a crucial component that affects the quality of sleep. Students who live in private accommodations and hostels are more likely to have poor sleep quality. Furthermore, sleep hygiene patterns, specifically the act of eating in bed or shortly before going to sleep, have been identified as significant factors that modified to improve the quality of sleep. Daytime sleepiness was correlated with many characteristics, such as being in a younger age group, pursuing non-medical academic disciplines, and engaging in substantial internet usage. Significantly, engaging in activities such as watching television, writing, and eating while in bed also played a role in increased daytime sleepiness.

## Appendices

Section A: socio-demographic characteristics

1. Age:

2. Sex: male/female

3. Batch:

4. Family income:

5. No. of Family Members:

6. Marital status:

7. Place of stay: home/hostel/private accommodation

8. Education of father: PG/ graduate/Higher Secondary/Secondary/Primary or below

9. Education of mother: PG/ graduate/Higher Secondary/Secondary/Primary or below

10. Do you use internet?    a) Yes                        b) No

11. On average how many hours do you spend online per day? ……….. hours

a. <1 hour  b. 1-2 hours  c. 2-3 hours  d. 3-4 hours  e. >4 hours

12. History of any known morbidities- Thyroid problems/Respiratory disorders/Cardiac disorders/any other (specify)

13. Are you on any medication? - YES/NO

If yes, specify

14. How will you rate your academic performance- Poor/Average/Excellent?

15. Have you been suffering from headache or low back ache or eye pain or heaviness of head in past 3 months? YES/NO

16. How much time is required for onset of sleep? (In minutes)

17. Do you feel Depressed? YES/NO

If Yes, How Often- daily/thrice weekly/weekly/monthly

Pittsburgh Sleep Quality Index

COMPONENT 1

During the past month, how would you rate your sleep quality overall?

very good (0)

Fairly good (1)

Fairly bad (2)

very bad (3)

COMPONENT 2

1. During the past month, how long (in minutes) has it usually taken you to fall asleep each night?

<15 minutes: 0,  16-30 minutes: 1,  31-60 minutes: 2,  >60 minutes: 3

2. During the past month, how often have you had trouble sleeping because you cannot get to sleep within 30 minutes.

Not during the past month: 0

Less than Once a week: 1

Once or twice a week: 2

*Three or more times a week: 3   *   

Assign component 2 score as follows:

            Sum of 1 and 2          Component 2 score

            1-2                                            1

            3-4                                           2

            5-6                                           3

COMPONENT 3

During the past month, how many hours of actual sleep did you get at night? (This may be different than the number of hours you spend in bed.)

HOURS OF SLEEP PER NIGHT

     Response                  score

     >7 hours                     0

     6-7 hours                    1

     5-6 hours                    2

     <5 hours                     3

COMPONENT 4

Number of hours slept-

Numbers of hours spent on bed-

Habitual sleep efficiency as follows: (Number of hours slept/Number of hours spent in bed) X 100 = Habitual sleep efficiency (%)

Assign component 4 score as follows:

  Habitual sleep efficiency %                       score

               >85%                                                 0

               75-84%                                              1

               65-74%                                              2

               <65%                                                 3

COMPONENT 5

During the past month, how often have you had trouble sleeping because you...

Have to get up to use the bathroom

Cough or snore loudly

Feel too hot

Feel too cold

Pain

Noise or light disturbances

Repeated night mares

Incomplete studies or work

Didn’t eat or drink during night

Not during the past 1 month- 0

Less than once a week- 1

once or twice a week- 2

three or more times a week- 3

Assign component 5 score as follows: Sum of all 9 problems

1-9                      1

10-18                  2

19-27                  3

COMPONENT 6

During past 1 month how often do you take medications

(over the counter drugs or prescribed)

Not during the past 1 month- 0

Less than once a week- 1

once or twice a week-  2

three or more times a week- 3

COMPONENT 7

A. During the past month, how often have you had trouble staying awake while driving, eating meals, or engaging in social activity?

Never- 0

Once or twice- 1

once or twice each week- 2

three or more times a week- 3

B. During the past month, how much of a problem has it been for you to keep up enough enthusiasm to get things done?

No problem- 0

only a very slight problem- 1

Some what a problem- 2

A very big problem- 3

Assignment Score = A+ B

0            0

1-2         1

3-4        2

5-6        3

Epworth Sleepiness Scale

**Table 4:** Epworth Sleepiness Scale 0: would never doze or sleep 1: slight chance of dozing or sleeping 2: moderate chance of dozing or sleeping 3: high chance of dozing or sleeping

Situation	Chance of dozing or sleeping			
	0	1	2	3
Sitting and reading				
Watching TV				
Sitting inactive in a public place				
Being a passenger in a car for an hour				
Lying down in the afternoon				
Sitting and talking to someone				
Sitting quietly after lunch (no alcohol)				
Stopping for a few minutes in traffic while driving				
Total Epworth score				

Sleep Hygeine Questionnaire

1. Do you read in bed? YES/NO

2. Do you watch TV in bed? YES/NO

3. Do you write in bed? YES/NO

4. Do you eat in bed? YES/NO

5. Do you eat prior to bedtime? YES/NO

SLEEP SCHEDULE

1. What time do you usually go to bed on weekdays?

0: <10 PM, 1: 10-11 PM, 2: 11 PM-12 AM, 3: >12 AM

2. What time do you usually get up on weekdays?

0: <6 AM, 1: 6-7 AM, 2: 7-8 AM, 3: >8 AM

3. What time do you usually go to bed on weekends?

0: <10 PM, 1: 10-11 PM, 2: 11 PM-12 AM, 3: >12 AM

4. What time do you usually get up on weekends?

0: <6 AM, 1: 6-7 AM, 2: 7-8 AM, 3: >8 AM

SLEEP PATTERN

How often do you snore? 0- Never 1-Daily 2- Weekly 3- Monthly

How loud is your snoring? 0- No snoring 1- Not very loudly 2- Somewhat loudly 3- Very loudly

## References

[1] (2023). Census 2011 India. https://www.census2011.co.in/.

[2] Steinberg L (2010). A behavioral scientist looks at the science of adolescent brain development. Brain Cogn.

[3] Colrain IM, Baker FC (2011). Changes in sleep as a function of adolescent development. Neuropsychol Rev.

[4] Peper JS, Brouwer RM, Schnack HG (2009). Sex steroids and brain structure in pubertal boys and girls. Psychoneuroendocrinology.

[5] Nelson KL, Davis JE, Corbett CF (2022). Sleep quality: an evolutionary concept analysis. Nurs Forum.

[6] Visvalingam N, Sathish T, Soljak M (2020). Prevalence of and factors associated with poor sleep quality and short sleep in a working population in Singapore. Sleep Health.

[7] Irish LA, Kline CE, Gunn HE, Buysse DJ, Hall MH (2015). The role of sleep hygiene in promoting public health: a review of empirical evidence. Sleep Med Rev.

[8] Yazdi Z, Loukzadeh Z, Moghaddam P, Jalilolghadr S (2016). Sleep hygiene practices and their relation to sleep quality in medical students of Qazvin University of Medical Sciences. J Caring Sci.

[9] Medic G, Wille M, Hemels ME (2017). Short- and long-term health consequences of sleep disruption. Nat Sci Sleep.

[10] Rezaei M, Khormali M, Akbarpour S, Sadeghniiat-Hagighi K, Shamsipour M (2018). Sleep quality and its association with psychological distress and sleep hygiene: a cross-sectional study among pre-clinical medical students. Sleep Sci.

[11] Basu M, Saha SK, Majumder S, Chatterjee S, Misra R (2019). A study on sleeping pattern among undergraduate medical students of a tertiary care teaching hospital of Kolkata. Int J Med Public Heal.

[12] Muralidhar M, Anaswara DU, Aiswarya E (2019). Sleep quality, its determinants and its association with academic performance among the students of a medical college in Kerala. Ann Community Heal.

[13] (2023). About the ESS - Epworth Sleepiness Scale. https://epworthsleepinessscale.com/about-the-ess/.

[14] (2012). STOP, THAT and One Hundred Other Sleep Scales. STOP, THAT One Hundred Other Sleep Scales.

[15] Mishra J, Panigrahi A, Samanta P, Dash K, Mahapatra P, Behera MR (2022). Sleep quality and associated factors among undergraduate medical students during Covid-19 confinement. Clin Epidemiol Glob Health.

[16] Nair RK, Ahmed M (2023). A study to assess the quality of sleep among medical students of Mysuru, Karnataka, India. Int J Community Med Public Health.

[17] Olmos IP, Delgado JM, González Reyes R, Talero Guitierrez C (2012). Sleep quality perception in youth population. Rev Cienc Salud.

[18] Paudel K, Adhikari TB, Khanal P, Bhatta R, Paudel R, Bhusal S, Basel P (2022). Sleep quality and its correlates among undergraduate medical students in Nepal: a cross-sectional study. PLOS Glob Public Health.

[19] Ramdhany YD, Devi Goorah SS, Cheeneebash J, Niketan Oodun R (2022). Factors associated with poor sleep among young people in Mauritius: a survey-based study. Int J Med Students.

[20] Sundas N, Ghimire S, Bhusal S, Pandey R, Rana K, Dixit H (2020). Sleep quality among medical students of a tertiary care hospital: a descriptive cross-sectional study. JNMA J Nepal Med Assoc.

[21] Corrêa CC, Oliveira FK, Pizzamiglio DS, Ortolan EV, Weber SA (2017). Sleep quality in medical students: a comparison across the various phases of the medical course. J Bras Pneumol.

[22] Shadzi MR, Salehi A, Vardanjani HM (2020). Problematic internet use, mental health, and sleep quality among medical students: a path-analytic model. Indian J Psychol Med.

[23] Schmickler JM, Blaschke S, Robbins R, Mess F (2023). Determinants of sleep quality: a cross-sectional study in university students. Int J Environ Res Public Health.

[24] Kaur G, Singh A (2017). Excessive daytime sleepiness and its pattern among Indian college students. Sleep Med.

[25] Giri P, Baviskar M, Phalke D (2013). Study of sleep habits and sleep problems among medical students of Pravara Institute of Medical Sciences Loni, Western Maharashtra, India. Ann Med Health Sci Res.

[26] Siddalingaiah HS, Mastin DF, Moore BD, Bryson WJ, D C, Singh A (2018). Prevalence and determinants of excessive daytime sleepiness among resident doctors at a tertiary care institution in India. Int J Community Med Public Heal.

[27] Alanazi EM, Alanazi AM, Albuhairy AH, Alanazi AA (2023). Sleep hygiene practices and its impact on mental health and functional performance among adults in Tabuk City: a cross-sectional study. Cureus.

[28] Dagnew B, Andualem Z, Dagne H (2020). Excessive daytime sleepiness and its predictors among medical and health science students of University of Gondar, Northwest Ethiopia: institution-based cross-sectional study. Health Qual Life Outcomes.

[29] Calhoun SL, Vgontzas AN, Fernandez-Mendoza J, Mayes SD, Tsaoussoglou M, Basta M, Bixler EO (2011). Prevalence and risk factors of excessive daytime sleepiness in a community sample of young children: the role of obesity, asthma, anxiety/depression, and sleep. Sleep.

[30] Singh LK, Suchandra KH, Pattajoshi A (2019). Internet addiction and daytime sleepiness among professionals in India: a web-based survey. Indian J Psychiatry.

